# Ultrafast Laser-Induced Surface Texturing to Enhance Stainless Steel Gliding on Snow

**DOI:** 10.3390/nano16120740

**Published:** 2026-06-13

**Authors:** Guglielmo Marchesa, Lorenzo Puppo, Matteo Verdi, Giorgia Dassiè, Federico Bassi, Etienne Negri, Enza Fazio, Enrico Gallus, Paolo Maria Ossi

**Affiliations:** 1 Dipartimento Ingegneria, Università degli Studi di Palermo, Viale delle Scienze Ed.8, 90128 Palermo, Italy; guglielmo.marchesa@unipa.it; 2 Dipartimento Energia, Politecnico di Milano, Via Lambruschini 4a, 20156 Milano, Italypaolo.ossi@polimi.it (P.M.O.); 3 Kirana S.r.l., Via Fortunato Zeni 8, Polo Meccatronica, 38068 Rovereto, Italy; f.bassi@kirana-laser.it (F.B.);; 4 Dipartimento Scienze Matematiche e Informatiche, Scienze Fisiche e Scienze della Terra, Università degli Studi di Messina, Viale Ferdinando Stagno d’Alcontres 31, 98166 Messina, Italy

**Keywords:** AISI 301H SS, snow, friction, surface properties, hydrophobicity, laser texturing, LIPSS

## Abstract

Ultra-High Molecular Weight Polyethylene (UHMWPE), the standard base material in ski manufacturing, offers excellent gliding performance but exhibits limited mechanical and scratch resistance on hard and icy snow conditions. In this work, stainless steel is proposed as a mechanically robust alternative, and its inherently higher friction against snow is addressed through surface engineering. The snow friction behavior of 301H stainless steel surfaces decorated with fishbone-like microstructures combined with Laser-Induced Periodic Surface Structures (LIPSSs) was investigated using a custom-built snow tribometer. Several pattern designs, with different pitch distances and depths, were engraved using femtosecond laser pulse irradiation. We conducted morphological, physical, and chemical investigations through microscopy, static contact angle measurements, and X-ray Photoelectron Spectroscopy analyses. Results indicate that the gliding performance is not directly related to the modifications in surface chemistry and wetting behavior of the samples but is affected by the geometry and orientation with respect to the sliding direction of the specific micro- and nano-features. Overall, we achieved friction coefficient values comparable to those found in UHMWPE with a fast and economically sustainable single-step laser-texturing process. This approach allows the industrial up-scaling of the fishbone-texture design to real-size alpine ski prototypes.

## 1. Introduction

Alpine skiing is characterized by a strong dependence, at both competitive and amateur levels, on the interaction between the base of the ski and the snow on which it glides. Bases of all commercially available skis are made of Ultra-High Molecular Weight Polyethylene (UHMWPE; for short, PE), with an average molecular weight of 2 to 6 million g mol^−1^ [1].

Since the 1960s, thanks to its excellent gliding properties on natural snow, this material has stood the test of time, contrary to the continuous innovation in construction materials and geometry of skis. Gliding performance is further enhanced by additives (waxes) developed for use in a wide range of environmental conditions, the most relevant factors being snow and air temperature, humidity, and wind. PE rolls with linear lengths of hundreds of meters are produced by a cost-effective extrusion process for lower-level skis and by sintering for high-performance skis [2]. During the final stage of PE production, the rate at which it naturally cools is crucial. The most valuable part of the rolls is the central section, which cools more slowly and thus shows the highest degree of polymer crystallization. This is associated with a volume reduction and the simultaneous formation of micro-voids, which are helpful in retaining the wax. The bases for top-level skis are obtained from the roll core of sintered PE. In addition to its outstanding processability, PE has the highest impact strength among polymers and a relatively high resistance to abrasion when gliding on natural snow. The very low friction coefficient on snow is further reduced by texturing the base surface with micro-grooves. The resulting surface pattern shows different geometries designed for various snow conditions. Carbon-based additives, such as graphite or carbon black, whose presence is revealed by the black color of the base, are dispersed throughout the PE matrix to further reduce the friction coefficient, making the skis faster by eliminating unwanted tribo-electricity contributions [1].

The suitability of PE for skiing is challenged primarily by the shift in the winter conditions that we have experienced in the last thirty years. A shorter natural snow season, widely confirmed by meteorological studies [3,4], has made artificial snow essential for guaranteeing acceptable slope conditions for the ever-increasing number of skiers [5]. This man-made snow, along with the intentionally high-density, icy surfaces prepared for competition, is significantly harder, more compact, and thus more abrasive than its natural counterpart [6]; as such, it results in important performance and durability limitations of PE bases. Upon gliding at high velocities on aggressive snow, the ski edges heat up and cause local heating of the PE near-surface regions adjacent to them up to temperatures above the curing temperature, resulting in irreversible material deterioration associated with the diffusion of the carbon-based additives onto the snow [1]. Aside from this, environmental concerns regard the release of microplastics in the snowpack, which occurs irrespective of the gliding velocity. The presence of PE was assessed in snow collected on the field, and the release of PE from the ski bases was proved through specific experiments in the laboratory [6]. It should be noted that one of the challenges worsened by the higher friction on hard artificial snow is the degradation of ski edges. Ski edges are made of low-carbon content steel. Moving from the above-described situation, in the last ten years research at the Politecnico di Milano was conducted to identify promising candidate materials to substitute for PE bases, aiming to construct the ski base and edges as a single component with better mechanical properties than PE, namely, wear and scratch resistance, avoiding also oxidation after prolonged contact with melted snow. AISI 301H stainless steel was found to offer a promising solution, with superior mechanical robustness and the possibility of constructing a unified base–edge. However, untreated steel suffers from a dramatic increase in friction attributed to capillary suction when gliding on snow that is undergoing accelerated metamorphism at temperatures near the melting point. To reduce snow friction by morphological and chemical modifications, laser-based techniques have been explored [7,8,9]. Indeed, hierarchical micro- and nano-laser surface texturing on different metals is associated with improved hydrophobicity and reduced ice friction in the hydrodynamic lubrication regime [10,11]. Nevertheless, ultra-short pulsed lasers have been employed in reducing friction in different oil-lubricated friction regimes for mechanical moving parts [12,13]. Furthermore, laser-induced bio-inspired surface features of microchannels and double-hierarchy roughness, including shark skin, fish scales, lotus leaf, and pitcher plant, have been shown to be able to reduce hydrodynamic drag friction in both laminar and turbulent flows under several flow configurations [14,15,16,17]. Supported by these encouraging results, in this work we present the combination of Laser-Induced Periodic Surface Structures (LIPSSs), as a promising stochastic (self-organized) solution, and a novel category of precisely engineered deterministic (grooved) surface structures. We discuss the results of tribological tests of different pattern designs and an analysis of their durability. The tribological performance is correlated with the underlying physicochemical surface modifications, and conclusions are drawn to identify the most robust and effective structure to improve stainless steel gliding properties. The optimized process parameters were transferred to the industrial production of full-scale ski prototypes.

## 2. Materials and Methods

### 2.1. Laser Processing

We fabricated the samples on a 50 mm × 50 mm × 0.5 mm AISI 301H steel sheets. The laser source used for the sample preparation was a 20 W Carbide CB3 (Light Conversion, Vilnius, Lithuania) with the fundamental wavelength of 1030 nm and pulse duration of 217 fs. The laser beam was focused on the substrate surface through an F-Theta lens (focal length 100 mm) and moved across the surface with a galvanometer scanner (ProSeries I 14 mm Cambridge Technology, Lexington, MA, USA). The laser spot size was set to 50 µm at the focal position through a manual beam expander, and the repetition rate was set to 1 MHz. To obtain a LIPSS superimposed on the deterministic microchannel pattern, the pulse energy was reduced to 4 µJ, and the beam was scanned over the entire sample area.

### 2.2. Surface Analysis

We morphologically analyzed the manufactured samples by white light interferometry (SmartWLI Compact white light interferometer, with a Nikon 20× Mirau objective GBS metrology GmbH, Ilmenau, Germany) and optical microscopy (Keyence VHX digital microscope, Osaka, Japan) to validate the microfeature geometry. We performed Scanning Electron Microscopy (SEM; Supra 40 field-emitting microscope operating at a primary accelerating voltage between 5 kV and 12 kV, from Zeiss, Oberkochen, Germany) analyses to detail the produced LIPSSs nanostructure. We analyzed the surface chemical composition by the X-ray Photoelectron Spectroscopy (XPS) technique with a limited sampling depth of about 10 nm. We used a Thermo Scientific K-Alpha spectrometer (Waltham, MA, USA) equipped with a monochromatic Al Kα X-ray source (1486.6 eV) and a hemispherical analyzer operating in constant analyzer energy mode (CAE). High-resolution (HR) spectra were acquired using a Pass Energy of 50 eV and an Energy Step of 0.1 eV. The instrumental contribution to the resolution, which scales with the Pass Energy, was kept sufficiently low to resolve the chemical shifts of interest. To maintain an optimal signal-to-noise ratio, we employed a Dwell Time of 100 ms and performed 5 scans for each region. The overall system performance was periodically calibrated against the Ag 3d_5/2_ peak to ensure consistency and precision. The survey spectra were collected with the following parameters: pass energy of 150 eV, energy step of 0.5 eV, number of scans and dwell time of 4 and 10 ms, respectively. We performed data processing and peak fitting using the Advantage software (v 5.9931) supplied with the K-Alpha system. Samples were cleaned in situ under vacuum by gently sputtering the surface with argon ions (3 keV, 60 s) to ensure the removal of adventitious carbon and other surface contaminants.

### 2.3. Contact Angle Analysis

We measured the static contact angle for the fabricated samples with a custom-built sessile drop goniometer setup and analysis software. We used a collimated white LED as the illumination source. We placed a telecentric Mitutoyo 1× (Mitutoyo, Kawasaki, Japan) coupled with an IDS high-resolution camera (IDS, Obersulm, Germany) on the same optical axis as the light source. We positioned the light source, the sample holder, and the sensor on the same rail to ensure optical alignment. We placed 10 µL droplets of deionized water droplets at the center of the sample using a calibrated micropipette. We moved the sample holder along the optical axis to ensure the droplet was at the focus of the objective. Once we captured the image of the droplet, we extracted the contact angle using internally developed software, which fitted the droplet edge contour and computed the CA as the angle between the tangent vectors at the three-phase contact line and the manually defined baseline corresponding to the sample surface. To account for the structural anisotropy introduced by the laser-induced surface features, measurements were performed both longitudinally and transversely with respect to the principal symmetry axis of the sample. Each reported CA value represents the average of these two orientations. For each orientation, the CA was determined as the mean of the left and right contact angles extracted from five successive analyses of the same sessile droplet.

### 2.4. Friction Tests

We conducted the experimental campaign using a custom-built laboratory snow tribometer designed to simulate a ski base sliding on a groomed slope. Our device consists of a rotating aluminum plate driven by a brushless electric motor, which houses an annular snow track prepared within a custom expanded polystyrene mold to ensure thermal insulation. To replicate realistic skiing conditions, the snow track consists of compacted layers: a bottom layer of hard ice to provide structural stability and a top layer of fine-grained snow to mimic a groomed surface. We performed tests under controlled environmental conditions, monitoring and maintaining the snow track temperature at −3 °C to target the critical snow-friction window in the hydrodynamic lubrication regime for metallic bases. Indeed, previous studies have shown that AISI 301H exhibits similar gliding performance to UHMWPE at lower temperatures (T = −5 °C, −10 °C) [10,11]. The snow temperature was monitored by a thermocouple inserted in the track at the beginning of each test and at the end of the same test. The typical test duration is 45 s. During this period, the track slowly warms up. The measured track temperature at the end of the test was between −2 °C and −1.5 °C. We mounted the samples on a dedicated holder and applied a normal load using a calibrated weight. We set the total mass acting on the sample to m = 0.692 kg, calculated to simulate the specific pressure exerted by a skier with mass 80 kg on a standard pair of alpine skis (length, 175 cm; average width, 8.5 cm). With a previously described procedure [9], we determined the friction coefficient (µ) via coast-down tests: the reported values were calculated as the average over the velocity range between 6 m s^−1^ and 0.6 m s^−1^. To compare different surface textures, we performed two subsequent tests for each sample under identical conditions, and we averaged between the two obtained µ values.

## 3. Results and Discussion

### 3.1. Pattern Design

We produced Fishbone (FB) grooved microstructures, whose main geometrical parameters are the angle, pitch, depth, and width of the grooves. The goal of this pattern is to mimic some surface patterning known to successfully reduce the friction of PE gliding on snow [18]. Accordingly, the produced microfeatures lie at a predetermined angle with respect to the sliding direction. Based on our study on the effect of FB angle orientation (0°, 10°, 45°, 60°, 70°, 90°) with respect to the snow sliding direction, we selected 45° as the optimum for further parametric optimization. In the first iteration of samples, we superimposed LIPSSs onto the previously engraved FB pattern, both parallel (LiPar) and orthogonal (LiOrt) to the sliding direction. We also produced in a single step a grooves-only control sample (NoLi). Based on the tribological results and industry-oriented considerations, which we present and discuss below (see Section 3.3), we focused on the deterministic grooves pattern only. Thus, in the second iteration, we studied the optimization of the micrometric FB geometry by varying the pitch and depth of the grooves, while maintaining the width fixed to 60 μm. We named all samples FBangle_pitch_depth_LIPSS. For simplicity, we assigned an Arabic number (1 to 6) to each sample. The reference AISI 301H untreated (bare) stainless steel sample is labeled as 0. Table 1 provides the full list of produced and tested samples along with their geometric pattern specifications. The textured area is the fraction percentage of the textured area (considering both micrometric and nanometric features) relative to the total surface area.

**Figure 1:**
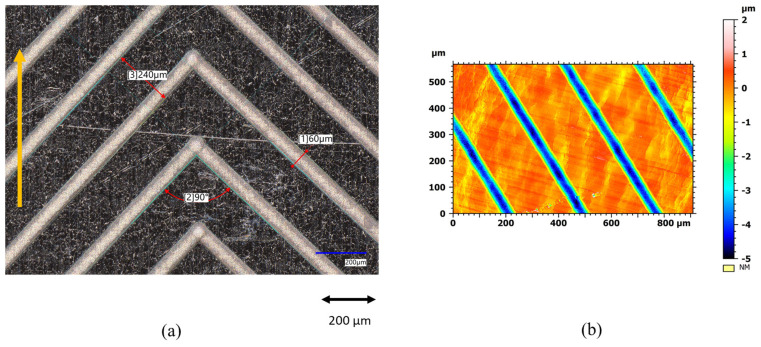
Fishbone microstructure: Digital microscope image (**a**) and interferometric analysis (**b**) of sample 6, showing the characteristic FB pattern, pitch distance, line width, and depth of textured area. The orange arrow in (**a**) indicates the sliding direction of the microstructures over the snow for the snow-friction tests.

**Figure 2:**
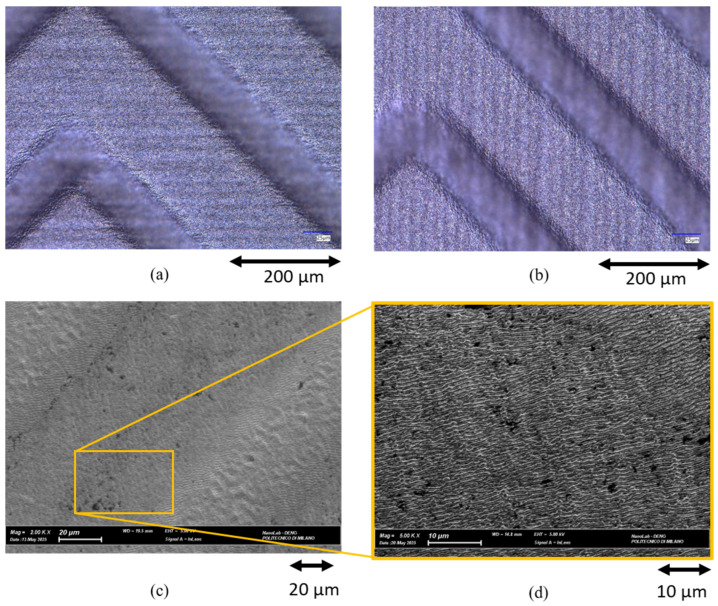
LIPSS orientation: Optical micrographs of samples 2 (**a**) and 3 (**b**), together with SEM micrographs of sample 2 taken at 2000× (**c**) and 5000× (**d**) magnification, showing LIPSS orientation with respect to the micrometric pattern.

Figure 1 displays a representative optical digital microscopy image of the manufactured samples, together with interferometric surface topography reconstruction. In Figure 2, both optical and SEM micrographs show LIPSS formation on the surface and demonstrate their alignment relative to the grooves.

### 3.2. Wettability and Surface Chemistry

**Figure 3:**
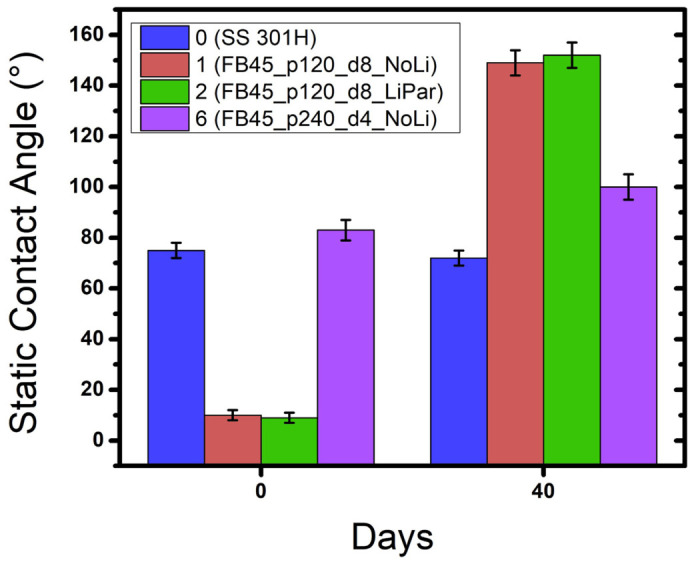
Static contact angle: Static contact angle values right after laser irradiation and after 40 days of air exposure, for samples 0, 1, 2, and 6.

We performed the static CA measurements right after laser fabrication (day 0) and after 40 days of ambient air exposure to allow the surface to reach a stationary wettability value. We compared the static CA of bare steel (AISI 301H) with that of FB samples with different pitch and depth (with and without LIPSS). For samples 0, 1, 2, and 6 we measured CA values of 75°, 8°, 9°, and 85° at day 0 respectively, and 75°, 150°, 148°, and 100° after 40 days (Figure 3). Our study confirms that the laser treatment drastically increases the surface wettability of bare steel right after fabrication, followed by a superhydrophobic transition over time [11]. This effect is particularly evident for samples where the textured area corresponds to at least 50% of the whole surface (see Table 1). Indeed, as the laser-textured area decreases to 25% for sample 6, no hydrophilicity is shown at day 0, while the CA after 40 days is slightly larger than that of bare steel (sample 0). At day 0, the laser-textured channels, intrinsically decorated with LIPSS, are highly reactive and hydrophilic, so the drop wets all the textured regions in all samples. In sample 2 (100% textured area), the droplet sits entirely on a hydrophilic surface, whereas in samples 1 and 6 only a fraction of the surface is textured (50% and 25%, respectively). Consequently, the lower textured fraction and shallower features (4 µm) of sample 6 are insufficient to influence its macroscopic wettability through chemical effects alone. In contrast, the larger textured fraction and deeper features (8 µm) of sample 1 provide enough liquid–solid contact area to affect macroscopic wettability. After 40 days, the textured regions develop hydrophobic behavior due to the formation of low-surface-energy oxide compounds, as confirmed by XPS analysis (see below). The sessile drop therefore sits in a Cassie–Baxter state on all samples. The combined effect of surface geometry and chemical composition governs the apparent macroscopic CA: sample 6 exhibits only partial hydrophobicity, while the effect is markedly stronger in samples 1 and 2, where both contributions are more significant, yielding higher contact angles.

As reported in Table 2, following the Ar^+^ ion etching step, the nitrogen content of the laser-treated sample drops sharply to 0.21 at.%, returning to a baseline value that is statistically identical to that of the etched bare steel (0.27 at.%). This behavior clearly demonstrates that the laser-induced nitrogen enrichment is strictly a surface phenomenon, confined to a topmost nanometric layer. Once this modified native layer is sputtered away, the pristine bulk chemistry of the steel is recovered, confirming that nitrogen did not diffuse deeply into the bulk substrate. Therefore, XPS quantitative analysis yields meaningful results only after Ar^+^ etching if one aims to realistically evaluate the true effects of the laser treatment on the bulk material. 

**Figure 4:**
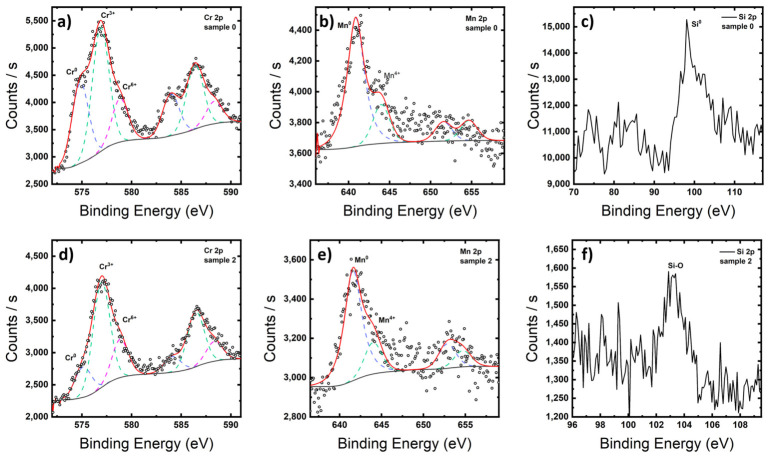
XPS lineshapes: XPS Cr 2p and Mn 2p high-resolution deconvoluted (**a**,**b**,**d**,**e**) and Si 2p (**c**,**f**) lineshapes. Circles and red lines refer to the experimental and deconvoluted profiles, respectively. Dashed lines refer to the fitting sub-bands.

The laser treatment induced a significant increase in oxygen content, rising from ~32 at.% on the bare steel (Sample 0) to ~49 at.% on the treated surface (Sample 2). This compositional restructuring was accompanied by a drastic redistribution of the metallic alloying elements. Specifically, a near-complete depletion of nickel was observed, with its concentration dropping sharply from ~5.7 at.% to 0.4 at.%. In contrast, the thermal process drove the surface segregation of manganese and silicon from the bulk material; manganese increased tenfold to ~4.9 at.%, while silicon tripled to 3.0 at.%. Given that the chromium concentration remained stable at approximately 11 at.%, these findings indicate that the laser processing transforms the conventional Cr-Ni passive film into a nickel-depleted, mixed-oxide layer highly enriched in both manganese and silicon. These observations are confirmed by the Cr 2p and Mn 2p lineshape fitting results (Figure 4 and Table 3) and the Si 2p lineshape change.

The Cr 2p lineshape is characterized by the Cr 2p3/2 (~574–580 eV) and Cr 2p1/2 (~583–590 eV) spin-orbit split doublet. Deconvolution of the Cr 2p envelope resolved three distinct chemical environments centered at approximately 575, 577, and 579 eV, which are assigned to metallic Cr (Cr^0^), trivalent chromium compounds (Cr_2_O_3_/Cr(OH)_3_), and hexavalent chromium oxide (CrO_3_), respectively. Following laser treatment, a significant enhancement in the chromium oxide phases was observed relative to the metallic component. Conversely, the Mn 2p spectrum exhibits a well-defined, wide spin-orbit splitting (Δ = 11.2 eV), with the relative proportions of metallic and oxidized manganese species remaining practically unaltered post-treatment. Notably, laser processing induced the formation of a silicon oxide phase, which was entirely absent in the pristine sample.

Stainless steels contain small amounts of silicon as an alloying element, typically ≤1 wt.%. Prior to laser processing, the bulk alloy is protected by a native passivation layer, primarily composed of chromium, with a shallow thickness of 1–3 nm. Within this ultra-thin surface region, the silicon signal is significantly diluted and heavily attenuated by the heavier matrix elements, placing it below the detection limit of conventional XPS. Following laser surface texturing, a distinct oxidized silicon phase emerges. During laser irradiation, the localized material undergoes rapid melting, vaporization, and plasma expulsion at extremely high temperatures. This extreme thermal cycle drives two simultaneous phenomena that enhance the silicon photoemission signal. First, thermal segregation and surface enrichment occur, whereby minor alloying elements, like silicon, diffuse rapidly toward the cooling surface, drastically increasing their surface concentration relative to the bulk. Moreover, due to the high reactivity of the molten phase in ambient air, the segregated silicon instantly bonds with atmospheric oxygen to form silicon oxide (predominantly SiO_2_), appearing as Si^4+^ in the XPS spectra. The concentration of this oxide within the top few nanometers of the surface renders it highly visible and easily detectable via XPS.

Simultaneously, the behavior of chromium (Cr), manganese (Mn), and nickel (Ni) undergoes a radical change driven by the extreme thermal shock and the distinct thermodynamic oxygen affinities of each element. XPS analysis resolves these variations primarily through thermal segregation and selective oxidation phenomena. On the pristine, untreated surface, the Mn XPS signal is nearly negligible or highly attenuated. Following laser treatment, a pronounced enhancement in the surface manganese intensity contributions is observed. Manganese possesses both a high vapor pressure (promoting rapid volatilization) and an extremely negative Gibbs free energy of oxide formation (MnO or Mn_3_O_4_). Concurrently, the relative percentage of chromium detected by XPS within the outermost surface layer remains unaltered. However, chromium is now positioned slightly deeper within the laser-induced mixed oxide film, becoming partially shielded from the surface-sensitive XPS analysis. Consequently, the detected chromium signal shifts almost entirely towards oxidized Cr species.

Finally, regarding nickel, this element exhibits a significantly lower oxygen affinity compared to Mn, Cr, and Fe, behaving as a noble species within this thermodynamic framework. During the laser-induced melting and subsequent solidification cycles, Ni is rejected by the advancing oxidation front. As a result, nickel concentrates in its metallic form within the underlying bulk, beneath the newly generated laser oxide layer. Because the thickness of this fresh oxide scale reaches several tens of nanometers, effectively exceeding the maximum sampling depth of XPS, the Ni photoelectrons are strongly attenuated and fail to reach the detector. Overall, the modified surface chemistry, characterized by the loss of hydrophilic Ni oxides and the growth of the low surface energy Si and Mn oxides, correlates with the observed stability of the surface hydrophobicity.

### 3.3. Snow Friction

**Figure 5:**
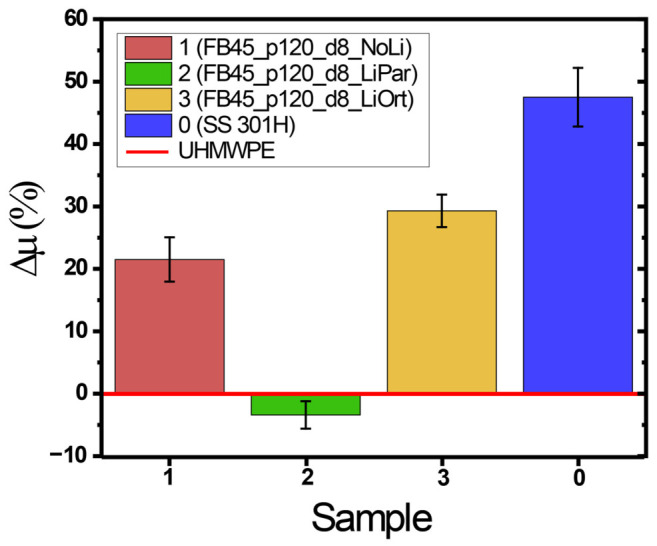
Effect of LIPSS orientation on the friction coefficient: Relative variation of friction coefficient Δμ with respect to the reference PE for samples 1, 2, 3, and 0 at T_snow_ = −3 °C.

We performed all friction tests on the snow tribometer under the most critical conditions for steel–snow friction, namely at a snow temperature of T = −3 °C [9]. Under this hydrodynamic lubrication regime, where the liquid water lubricating film is thicker than the surface asperities, hydrodynamic and capillary drag represent the major sources of friction [10]. In Figure 5, we present the variation of the friction coefficient from FB-textured samples produced in the first iteration (see Section 3.1) with respect to PE, with LIPSSs oriented parallel (sample 2) and orthogonal (sample 3) to the sliding direction, together with FB-textured surface without LIPSSs (sample 1) and bare steel (sample 0). We tested all samples with the arrow-like pattern pointing towards the sliding direction (see Figure 1).

In the hydrodynamic lubrication regime, the FB microstructure alone efficiently reduces the coefficient of friction of bare AISI 301H steel on snow. Indeed, the FB-textured samples without LIPSSs (sample 1) showed a coefficient of friction 21% larger than PE, but still over 50% smaller than sample 0. We attribute this result to a combination of different mechanisms. On the one hand, the reduction by 50% of the apparent contact area interacting with the snow substrate (see Table 1) leads to a proportionally reduced frictional adhesive force. Aside from this, the hydrophobic surface chemistry (see Section 3.2) guarantees a stable Cassie–Baxter wetting state with consequent air entrapment in the surface microfeatures [10]. However, these mechanisms are related to systems in a static domain, while the dynamic nature of snow gliding allows to take advantage of the characteristic orientation of the FB microstructure in the hydrodynamic flow regime. Indeed, for flat SS surfaces the melt-water film produced by frictional heating remains trapped in between the snow and the flat, untreated surface of bare steel (sample 0). The resulting capillary bridges between them generate suction-like drag forces. On the contrary, within the FB features, the water layer is broken-up, and the water is channeled away by the 45° inclined channels which act as escape paths. This allows for the consequent reduction of the water film thickness, which reaches its optimum value in the mixed-lubrication regime [10,18]. We previously demonstrated [9] how the most critical condition for steel gliding on snow coincides with snow temperatures close to melting. In such conditions, the hydrodynamic regime is likely to govern friction, with the liquid water film becoming too thick. Evidently, it is required to laterally eject water outside the system.

We also noted the strong influence on gliding performance of the specific orientation of the produced surface nano-morphology. FB-textured samples without LIPSSs (sample 1) performed in between the parallel (sample 2) and orthogonal (sample 3)-oriented LIPSS. In the case of LIPSS-decorated samples, the coefficient of friction difference from that of PE drops from +29% to −3% when we changed the ridge orientation from perpendicular to parallel to the sliding direction. This result suggests a strong influence of feature orientation on the surface hydrodynamic drag behavior in the nanoscale domain. With LIPSSs parallel to the sliding direction, the meltwater is freely channeled, lowering viscous drag within the hydrodynamic film. Instead, when LIPSSs are oriented orthogonally, a higher resistance arises due to a physical barrier effect. This is not surprising, since in both laminar and turbulent hydrodynamic flow regimes micro-sized features aligned parallel to the flow direction reduce hydrodynamic drag more than the ones aligned orthogonal [19]. Although we found a similar trend, further analysis is needed to validate the hypothesis of scalable mechanisms from the micro- to the nano- scale. Up to now, the design of nanostructured surface patterns to reduce hydrodynamic drag focused solely on random nano-textures, without accounting for any preferred nano-orientation [20].

In both LIPSS configurations, the hydrophobic surface chemistry resulting from Mn and Si oxides (see Section 3.2), combined with the surface morphology, guarantees the Cassie–Baxter state responsible for the reduced snow-friction with respect to bare AISI 301H. Furthermore, the geometry of secondary surface features allows to explain the enhanced drag reduction that we observe for surfaces with hierarchical roughness compared with theoretical calculations, which only consider micro-sized features (e.g., sample 2 with respect to sample 1). Indeed, the secondary structures likely contribute to increase the amount of trapped air, corresponding to an increased effective slip length to reduce drag in the hydrodynamic flow regime [21]. The nano- and micro- grooves can firmly trap air only if the structure is supported by a sufficiently hydrophobic surface to resist water infiltration into the cavities. On a hydrophilic surface, the liquid meniscus would penetrate the features with consequent transition into the Wenzel state, thus removing any friction reduction. Chemical and morphological modifications act in a synergistic way to reduce the overall metal–snow friction.

Even though the FB with parallel-oriented LIPSSs (sample 2) showed the best gliding performance, we selected the FB without superimposed nanostructure (sample 1) for further parametric optimization because it can be produced in a single-step process rather than in two steps, providing a good balance between performance and processability of full-size ski bases.

**Figure 6:**
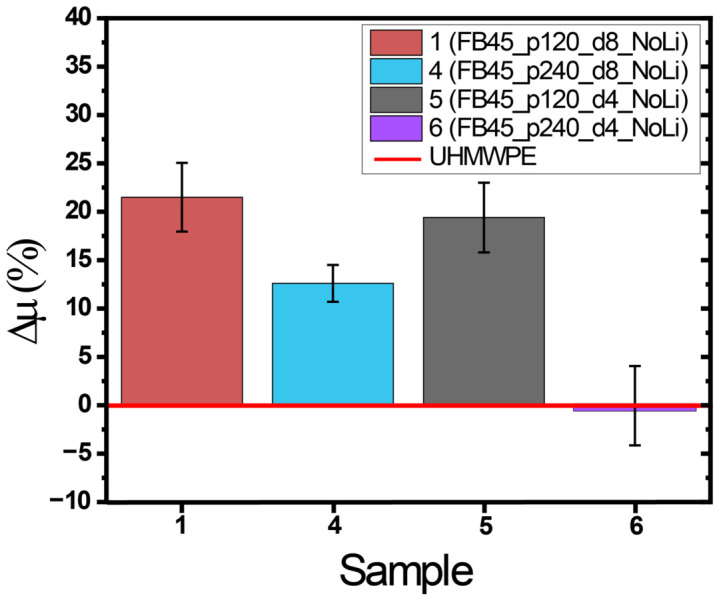
Effect of pitch and spacing on the friction coefficient: Relative variation of friction coefficient with respect to PE for samples 1, 4, 5, and 6 at T = −3 °C.

Aiming to further improve the snow-gliding ability of the FB microstructure without LIPSS post-processing, we varied the pitch distance and groove depth, defining the second iteration of samples (see Section 3.1). Snow tribometer tests, displayed in Figure 6, show that snow-gliding benefits from both an increased pitch distance (from 120 µm to 240 µm) and a shallower feature depth (from 8 µm to 4 µm), with the coefficient of friction respectively +13% and +19% higher than PE. Combining the above pitch and depth values (sample 6), the gliding performance turns slightly better than PE.

We attribute the improved gliding performance in samples with greater pitch distance to more stable air pockets trapped inside the microfeatures. Thanks to a favorable alternation of hydrophobic and hydrophilic strips along the surface (in our samples, the textured and untextured areas, respectively), the three-phase surface tension equilibrium (liquid–solid–gas) is more effective in stabilizing air bubbles inside the microfeatures [22].

Specifically, while the untextured area retains the hydrophilic chemical surface properties of bare AISI 301H, within the textured groove area, a stable hydrophobicity results upon the formation of Si and Mn oxides, as observed by XPS. The steep surface energy gradient between the two areas anchors the three-phase contact line in place, with consequent pinning of the water meniscus and stabilization of air pockets. In samples with greater pitch distance, the larger hydrophilic area, reflected in a static CA approaching that of bare AISI 301H (Figure 3), distributes the meltwater film over a wider untextured region, reducing the local hydrostatic pressure acting on the three-phase pinning force. This shifts the Cassie-to-Wenzel transition towards more severe external pressure conditions, consistent with the well-established principle that resisting the inward displacement of the contact line is the key challenge in maintaining stable Cassie-state hydrophobicity [23,24,25].

Deeper features further reinforce this resistance, as the water meniscus must travel a longer path along the feature walls to fully displace the air inside the pocket [26]. However, under the high external pressures typical of snow-gliding conditions, a full Cassie-to-Wenzel transition is likely unavoidable regardless of feature depth. In this regime, the relevant figure of merit shifts from wetting stability to hydrodynamic drag reduction, and shallower features prove advantageous. To efficiently reduce drag in such surfaces it is of crucial importance to optimize the feature geometry and spatial distribution depending on the specific flow conditions. This can be achieved by reducing the interface area between the substrate and the generated turbulent flow vortices, by forcing them to interact only with the apparent surface area, namely above the surface features. Similar mechanisms of fluid-drag reduction have been observed and replicated in several bio and bio-inspired structures (e.g., shark-skin riblets, fish scale, rice leaf, and butterfly wings) [27,28,29]. While deeper grooves favor the recirculation of flow currents inside the features with consequent increase of viscous drag, shallower features do not provide enough space to the vortices to fill the gaps, thus constraining them on the surface tips where less surface is available to generate drag friction. In summary, greater pitch and deeper features both contribute to Cassie-state stability, but once the Wenzel transition is pressure-induced, shallower features become hydrodynamically favorable. These two effects are therefore not contradictory, but complementary, each dominating under different conditions. The larger pitch distance and shallower features of sample 6 with the combination of their effects results in gliding properties comparable to those of PE, with negative relative variation Δμ of friction coefficient by –0.5% (Figure 6). The extremely low μ value of PE gliding on snow which results from the low surface energy of its hydrocarbon bulk chemical structure is achieved in AISI 301H by the combined effect of surface morphology and chemistry optimization.

**Figure 7:**
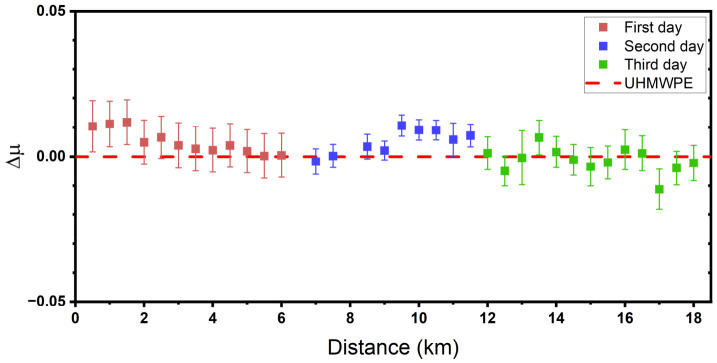
Long-run tests: Average difference of coefficient of friction relative to PE measured on the snow tribometer for sample 6 during repeated long-run tests at T = –3 °C.

As a last step, we selected the best-performing surface structure of sample 6 for long-run snow tribometer durability tests and full-scale ski prototyping. Under the same ambient conditions we used for all the above-discussed tribometer tests, we performed long-run tests by repeating the μ measurement along 36 successive runs, each covering an average distance of 500 m, for up to a total of 18 km. Further to visual inspection of the track, to verify that its surface was not damaged, after every single run the track was kept in the freezer, recording its temperature, for enough time to restore the snow track temperature at the desired value (−3 °C). In Figure 7, we display for each test the average difference of friction coefficient relative to PE. The data lie consistently within the range of previously collected data for sample 6. This means that the snow-gliding behavior of the treated surface remains stable over time. SEM images taken before and after long-distance tests showed that LIPSS surfaces are not morphologically altered through durability tests [9]. The same holds for a more robust and stable microstructure, such as that of FB features.

Adopting the same geometry (length, side-cut) as a commercial model, a prototype full-size ski pair with AISI 301H base was constructed and subjected to the same laser treatment as sample 6. The structure of such skis was slightly modified to take into account the difference in longitudinal and torsional resistance provided by steel, compared to PE. The skis were compared with a pair of skis of the same model, bearing standard PE bases, grinded.

Three professional testers compared the performance (gliding velocity, maneuverability) of skis with different base material and surface treatment following the experimental protocol described in detail elsewhere [30]. During free skiing tests with different ambient conditions, including low (down to −13 °C) and high (up to −1 °C) snow temperature, no appreciable differences were observed. In addition to this, we performed gliding tests, again comparing grinded, waxed state-of-the-art PE bases with laser-treated, unwaxed AISI 301H bases. The tests were conducted on a 60 m long, rectilinear track with a gentle, constant slope of about 10°. A starting gate and a photocell were mounted to record the time required to glide along the track without using the poles at the start time and avoiding any voluntary movement of the tester during the ski descent. We deliberately chose ambient conditions adverse to the performance of metallic bases: cloudy weather, air temperature between −1.2 °C and +0.4 °C, RH between 93% and 88%. After a faster acceleration of the waxed skis in the initial 2–3 m the two kinds of base performed the same way. We attribute this difference to the wax effect, which is known to impart a superhydrophobic wetting state to PE with very low contact angle hysteresis [31]. Indeed, waxed PE exhibits overall better gliding performance on snow than laser-treated stainless steel, though this advantage is macroscopically significant only at low speeds (below 3 m s^−1^). At higher speeds, the micro-structured surface pattern becomes dominant, as it channels away excess water generated at the lubricating layer, making the performance difference due only to the wettability behavior negligible [32,33]. The average running time (five runs) of skis with the PE base is 11.65 s, against 12.48 s of skis with the AISI 301H laser-treated base.

## 4. Conclusions

We textured FB micropatterns with a 45° angle on 301H stainless steel surfaces using pulsed femtosecond laser irradiation. By snow-friction tribometer testing, we observed improved gliding performance compared with bare steel surfaces. FB patterns with superimposed LIPSSs parallel and orthogonal to the sliding direction exhibited coefficients of friction of −3% and +29%, respectively, compared to the PE currently used for ski bases. Since both samples show comparable superhydrophobic behavior, our results highlight the role of feature orientation, combined with wettability, in the snow friction mechanisms.

Increasing the pitch distance and reducing the depth of FB microfeatures, without superimposing LIPSS, further enhances the surface gliding ability of AISI 301H, making the laser processing faster and more cost-effective. Under these conditions, the friction coefficient decreases from +21% to –0.5% relative to that of PE. This result was confirmed in long-run tribometer tests and field tests using full-size skis with laser-treated bases showing stable and repeatable performance. In conclusion, we demonstrated that FB micropatterns effectively reduce the friction coefficient of AISI 301H stainless steel on snow. The optimized pattern can be scaled over large areas in industrial applications, opening the way for real-world alpine ski texturing.

## Figures and Tables

**Table 1:** Sample nomenclature and details of the surface features. The symbols // and ⊥ stand respectively for parallel and perpendicular LIPSS orientation with respect to the sliding direction.

Sample	Name	Pitch [μm]	Depth [μm]	LIPSS	Textured Area [%]
1	FB45_p120_d8_NoLi	120	8	no	50
2	FB45_p120_d8_LiPar	120	8	//	100
3	FB45_p120_d8_LiOrt	120	8	⊥	100
4	FB45_p240_d8_NoLi	240	8	no	25
5	FB45_p120_d4_NoLi	120	4	no	50
6	FB45_p240_d4_NoLi	240	4	no	25

**Table 2:** Atomic species percentage of bare (0) and a representative laser-treated stainless steel (2), determined by XPS analysis. This latter was performed for both the un-modified and Ar+ ion-etched surfaces.

Element		Atomic%			
	Sample	0	0_etched	2	2_etched
C		82.57	48.23	63.12	31.52
O		14.19	32.54	29.33	48.97
N		0.13	0.27	5.01	0.21
Ni		-	5.72	-	0.38
Cr		1.66	11.19	0.55	10.77
S		0.49	0.35	0.19	-
P		-	0.28	0.25	0.22
Si		0.96	0.97	1.55	3.00
Mn		-	0.46	-	4.93

**Table 3:** Cr and Mn bonding fraction determined by XPS analysis.

	Cr^0^/Cr-N (%)	Cr^3+^ (Cr_2_O_3_)/Cr(OH)_3_ (%)	Cr^6+^ (CrO_3_) (%)
SS 301H (sample 0)	32.5	50.2	17.3
FB45_p120_d8_LiPar (sample 2)	17.5	60.8	21.7
	**Mn^0^ (%)**	**Mn^4+^ (MnO_2_) (%)**	
SS 301H (sample 0)	79.1	20.9	
FB45_p120_d8_LiPar (sample 2)	80.1	19.9	

## Data Availability

Data are available from the corresponding author upon reasonable request.
